# Supplementary material for: Morphofunctional Investigation in a Transgenic Mouse Model of Alzheimer’s Disease: Non-Reactive Astrocytes Are Involved in Aβ Load and Reactive Astrocytes in Plaque Build-Up
Source: Cells. 2023 Sep 12;12(18):2258. doi: 10.3390/cells12182258 (PMC10526848; doi:10.3390/cells12182258)
Supplement: Supplementary file 1 [file cells-12-02258-s001.zip › Legends Of Supplementary Flies.pdf]

## Legends of supplementary figures

Supplementary figure S1. Morphological integrity of the astrocyte meshwork in the transgenic mouse hippocampus: Sholl analysis. Sholl analysis were performed on representative astrocytes from WT-m (A), and 2m- (B), 6m- (C) and 12m- (D) Tg-m, stained to reveal GFAP by using the ImageJ plugin "Simple Neurite Tracer". Z-projections of 3D confocal stacks (gray) were merged with colorimetric rendering of the number of intersections between Sholl's spheres and the highlighted APJs. Bars = 15 μm.

Supplementary Figure 2. Acquisition parameters of autofluorescence and GFAP immunofluorescence in the hippocampus of transgenic mice. The diagram shows in two sequential scans the emission spectra of alexa488 (blue and cyan lines) fluorescence and autofluorescence (solid gray and dotted black lines) excited by the 458 nm (blue and solid gray lines) and 488 nm (cyan and dotted black lines) laser lines. In the first scan, the overlapping fluorescences were acquired in two different spectral windows by using the 458 nm laser line. In the spectral window between 495 and 252 nm (495-525l458, magenta area) the contribution of alexa488 emission was greater than the contribution of autofluorescence. In contrast, the degree of contribution of the two fluorescences was reversed in the spectral window between 560 and 590 nm (560-590l458, green area). The magenta and green color scales were assigned to the 495-525l458 and 560-590l458 channels, respectively. In the merged figures, autofluorescent deposits (green) can be discriminated from the immunostained processes of astrocytes (gray). In the second scan, the 488 nm laser line was used to maximize contribution of the alexa488 fluorescence (yellow area) acquired in the spectral window between 560 and 590 nm (560-590l488).

Supplementary figure S3. Relationships between APJs and amyloid plaques in the hippocampus of transgenic mice. CA1 hippocampal sections of 12m-Tg-m were immunostained for GFAP (cyan) to reveal APJs. Autofluorescence of Aβ-peptides was also detected (magenta). A) Autofluorescence in the CA1 hippocampus of 12m-Tg-m; the dotted line indicates the border between *stratum pyramidale* and *stratum radiatum*. B) Magnification of the framed area in A, showing interactions of the APJ meshwork with a cluster of amyloid plaques surrounded by small autofluorescent deposits; panels b1-b4 are details of the framed areas in B, which shows merging of small deposits and amyloid plaques (magenta channel, left), and their interactions with APJs (magenta and cyan channels, right). Bars: A = 40 μm; B = 20 μm; b1-b4 = 5 μm.

Supplementary figure S4. Dendrite fragmentation in the hippocampus of transgenic mice: AIVIA analysis. Fragments of MAP2+ dendrites were isolated and quantified using the AIVIA software in WT-m (A) and 2m-Tg-m (B), and in peri-plaque (D, F) and non plaque (C, E) domains of 6m-Tg-m (C, D) and 12m-Tg-m (E, F). Left panels shows MAP2 immunofluorescence (magenta) and amyloid plaques (blue); middle panels show intact dendrites (magenta) and fragments (yellow) discriminated by the AIVIA software; right panels show fragments isolated as objects by the AIVIA software. Bars = 14 μm.

## Legends of supplementary movies

Supplementary movie S1. 3D volume rendering of APJs from an astrocyte in the CA1 hippocampus of WT-m. APJs were traced and isolated with SNT, and then merged with color scale renderings of Sholl's measurements.

Supplementary movie S2. 3D volume rendering of APJs from an astrocyte in the CA1 hippocampus of 2m-Tg-m. APJs were traced and isolated with SNT, and then merged with color scale renderings of Sholl's measurements.

Supplementary movie S3. 3D volume rendering of APJs from an astrocyte in the CA1 hippocampus of 6m-Tg-m OUT. APJs were traced and isolated with SNT, and then merged with color scale renderings of Sholl's measurements.

Supplementary movie S4. 3D volume rendering of APJs from an astrocyte in the CA1 hippocampus of 12m-Tg-m OUT. APJs were traced and isolated with SNT, and then merged with color scale renderings of Sholl's measurements.

Supplementary movie S5. AIVIA 3D views of autofluorescent deposits and APJs in WT-m. From 0 sec to 20 sec: autofluorescent deposits (green) and GFAP immunostaining (gray) are shown in merged 495-525l458 and 560-590l458 channels. From 20 sec to 38 sec: classified deposits are shown in magenta together with autofluorescent deposits (green) and GFAP (gray). From 38 sec to 52 sec: classified deposits are shown by 3D volume rendering in purple color together with autofluorescent deposits and GFAP (560-590l488 channel, yellow). From 52 sec to 60 sec: classified contact sites (blue) between autofluorescent deposits (green) and APJs (gray). From 61 sec to 71 sec: Classified deposits associated with APJs are shown in cyan together with classified contact sites (blue) and GFAP (gray). From 71 sec to the end of the video: deposits associated with APJ are shown by 3D volume rendering in dark green color together with GFAP immunostaining (yellow). Apple operative systems will need either VLC (https://www.videolan.org/vlc/) or Elmedia video player (on AppleStore) to play this clip.

Supplementary movie S6. AIVIA 3D views of autofluorescent deposits and APJs in 12-m-Tg-m. From 0 sec to 20 sec: autofluorescent deposits (green) and GFAP immunostaining (gray) are shown in merged 495-525l458 and 560-590l458 channels. From 20 sec to 38 sec: classified deposits are shown in magenta together with autofluorescent deposits (green) and GFAP (gray). From 38 sec to 52 sec: classified deposits are shown by 3D volume rendering in purple color together with autofluorescent deposits and GFAP immunostaining (560-590l488 channel, yellow). From 52 sec to 58 sec: classified contact sites (blue) between autofluorescent deposits (green) and APJs (gray). From 58 sec to 64 sec: Classified deposits associated with APJs are shown in cyan together with classified contact sites (blue) and GFAP (gray). From 64 sec to the end of the video: deposits associated with APJ are shown by 3D volume rendering in dark green color together with GFAP (yellow). Apple operative systems will need either VLC (https://www.videolan.org/vlc/) or Elmedia video player (on AppleStore) to play this clip.

Supplementary movie S7. AIVIA 3D views of the double Cx43+GFAP immunostaining in a normal vessel of a WT-m. From 0 sec to 20 sec: immunostaining of GFAP is shown in cyan and immunostaining of Cx43 is shown in yellow. From 20 sec to 35 sec: clusters associated with APJ are shown in magenta together with GFAP (cyan) and Cx43 (yellow) immunostaining. From 35 sec to 60 sec: immunostaining of GFAP and Cx43 are excluded. Clusters associated with APJ are shown first in magenta and then by 3D volume rendering in cyan. From 60 sec to the end of the video: clusters associated with APJ are excluded from visualization and immunostaining of GFAP is shown in cyan and immunostaining of Cx43 is shown in yellow. Apple operative systems will need either VLC (https://www.videolan.org/vlc/) or Elmedia video player (on AppleStore) to play this clip.

Supplementary movie S8. AIVIA 3D views of the double Cx43+GFAP immunostaining in perivascular gliosis of a 12-m-Tg-m. From 0 sec to 20 sec: immunostaining of GFAP is shown in cyan and immunostaining of Cx43 is shown in yellow. From 20 sec to 35 sec: clusters associated with APJ are shown in magenta together with GFAP (cyan) and Cx43 (yellow) immunostaining. From 35 sec to 60 sec: immunostaining of GFAP and Cx43 are excluded. Cx43 clusters associated with APJ are shown first in magenta and then by 3D volume rendering in cyan. From 60 sec to the end of the video: clusters associated with APJs are excluded from visualization and immunostaining of GFAP is shown in cyan, immunostaining of Cx43 is shown in yellow. Apple operative systems will need either VLC (https://www.videolan.org/vlc/) or Elmedia video player (on AppleStore) to play this clip.

Supplementary movie S9. AIVIA 3D views of the double Cx43+GFAP immunostaining in peri-plaque glial scar in a 12-m-Tg-m. From 0 sec to 20 sec: immunostaining of GFAP is shown in cyan and immunostaining of Cx43 is shown in yellow. From 20 sec to 35 sec: clusters associated with APJ are shown in magenta together with GFAP (cyan) and Cx43 (yellow) immunostaining. From 35 sec to 60 sec: immunostaining of GFAP and Cx43 are excluded. Cx43 clusters associated with APJ are shown first in magenta and then by 3D volume rendering in cyan. From 60 sec to the end of the video: clusters associated with APJs are excluded from visualization, immunostaining of GFAP is shown in cyan and immunostaining of Cx43 is shown in yellow.

Apple operative systems will need either VLC (https://www.videolan.org/vlc/) or Elmedia video player (on AppleStore) to play this clip.

Supplementary movie S10. 3D volume rendering of APJs (cyan) enveloping the portion of a large plaque (magenta) included in the thickness of a CA1 hippocampal section of a 12m-Tg-m.

Supplementary movie S11. 3D volume rendering of APJs (cyan) enveloping a small plaque (magenta) totally embedded in the thickness of a CA1 hippocampal section of a 12m-Tg-m.

Supplementary movie S12. 3D volume rendering of panel b1 in Supplementary Fig. 3, showing the interactions of APJs (cyan) with small amyloid deposits and plaques (magenta) as they are merging.

Supplementary movie S13. 3D volume rendering of panel b4 in Supplementary Fig. 3, showing the interactions of APJs (cyan) with small amyloid deposits and plaques (magenta) as they are merging.

Supplementary movie S14. 3D volume rendering from panel b2 in Supplementary Fig. 3, showing APJs (cyan) stretching out from the surface of an amyloid plaque and contacting a neighboring deposit (magenta).

Supplementary movie S15. 3D volume rendering from panel b2 in Supplementary Fig. 3, showing APJs (cyan) stretching out from the surface of an amyloid plaque and contacting a neighboring deposit (magenta).

Supplementary movie S16. 3D volume rendering from panel b3 in Supplementary Fig. 3, showing APJs (cyan) stretching out from the surface of an amyloid plaque and contacting a neighboring deposit (magenta).
