# Supplementary figures and images for: Morphofunctional Investigation in a Transgenic Mouse Model of Alzheimer’s Disease: Non-Reactive Astrocytes Are Involved in Aβ Load and Reactive Astrocytes in Plaque Build-Up
Source: Cells. 2023 Sep 12;12(18):2258. doi: 10.3390/cells12182258 (PMC10526848; doi:10.3390/cells12182258)

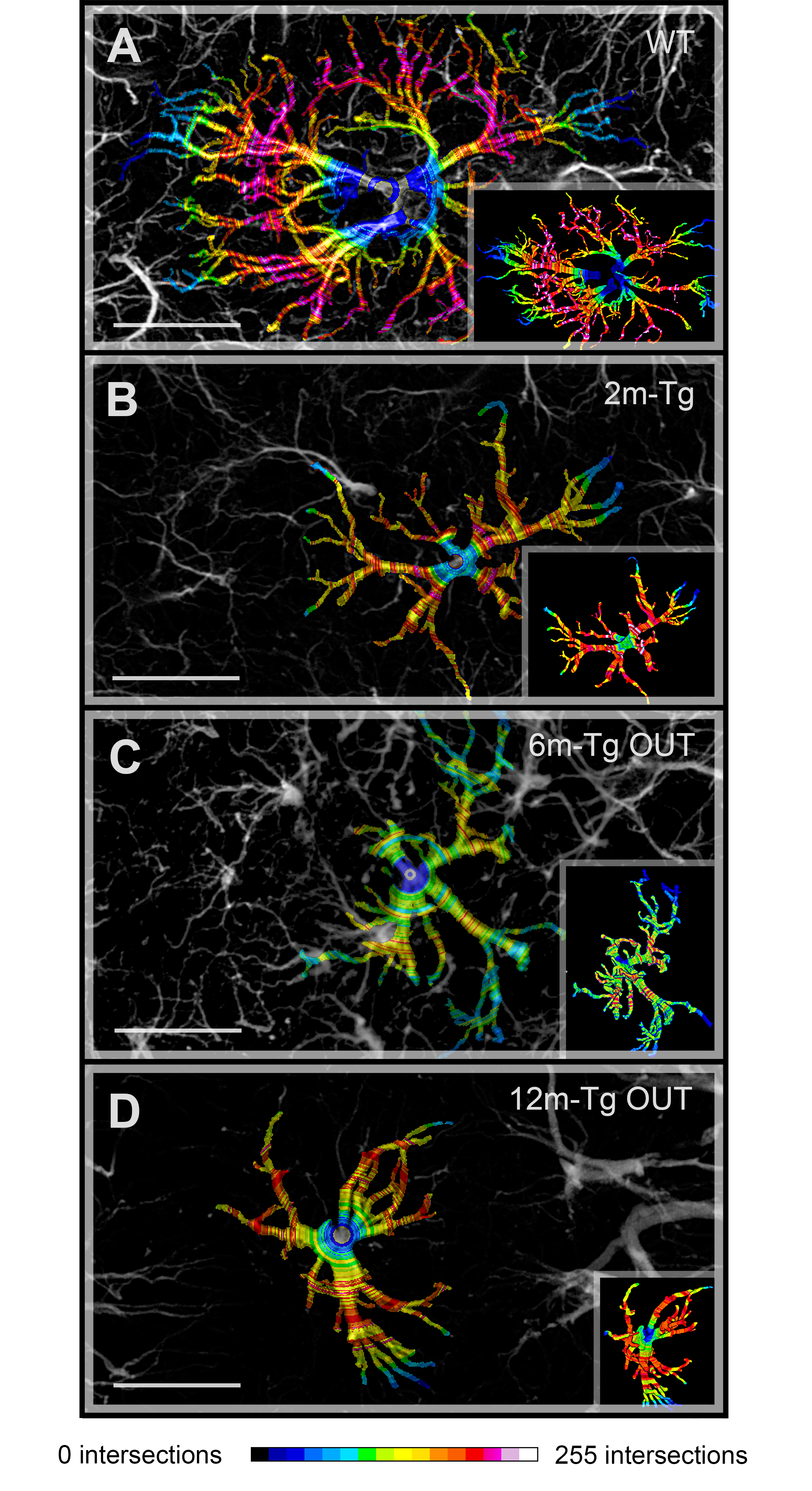

Supplement: Supplementary file 1 [file cells-12-02258-s001.zip › Supplementary figure S1.tif]

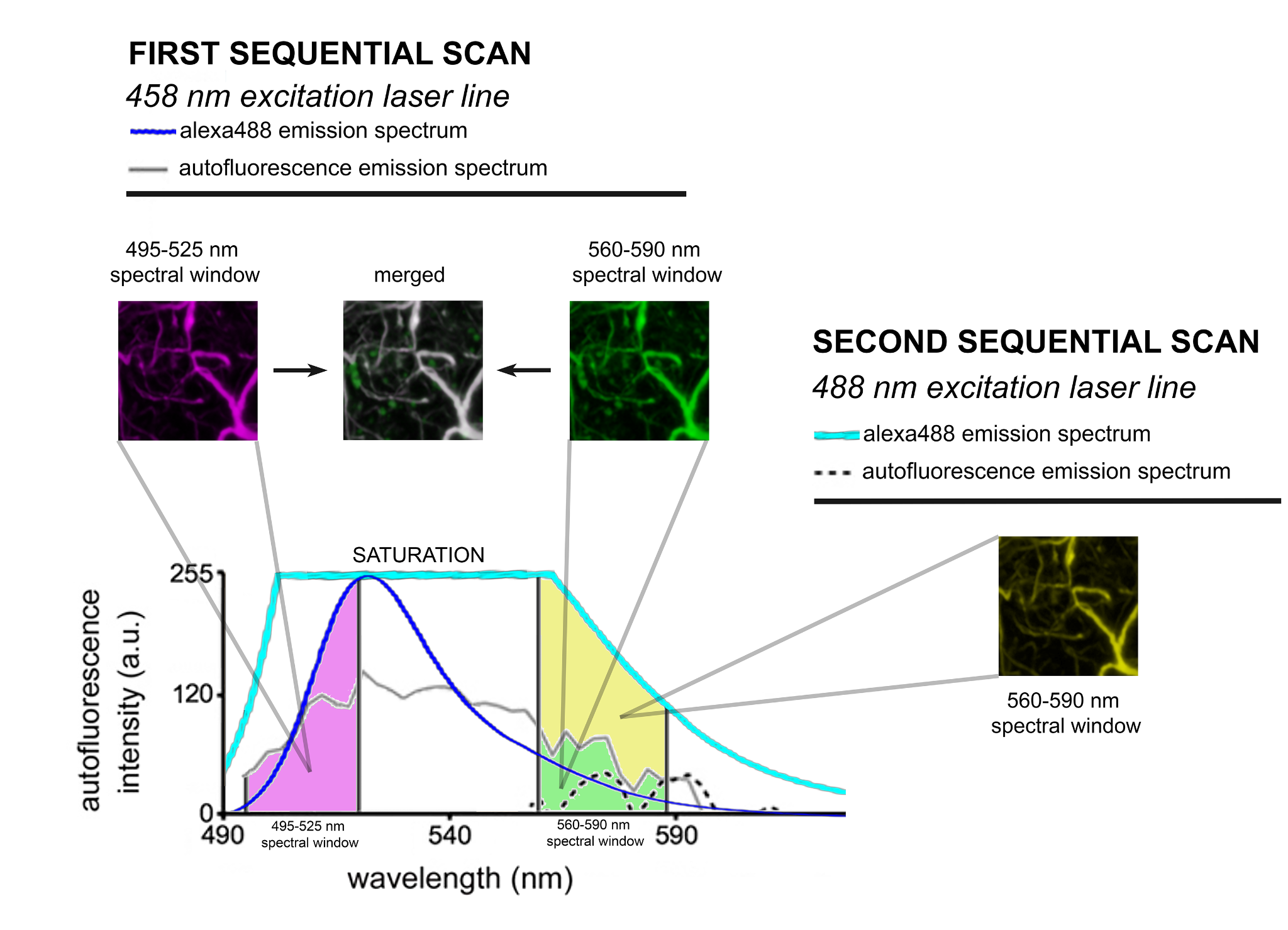

Supplement: Supplementary file 1 [file cells-12-02258-s001.zip › Supplementary figure S2.png]

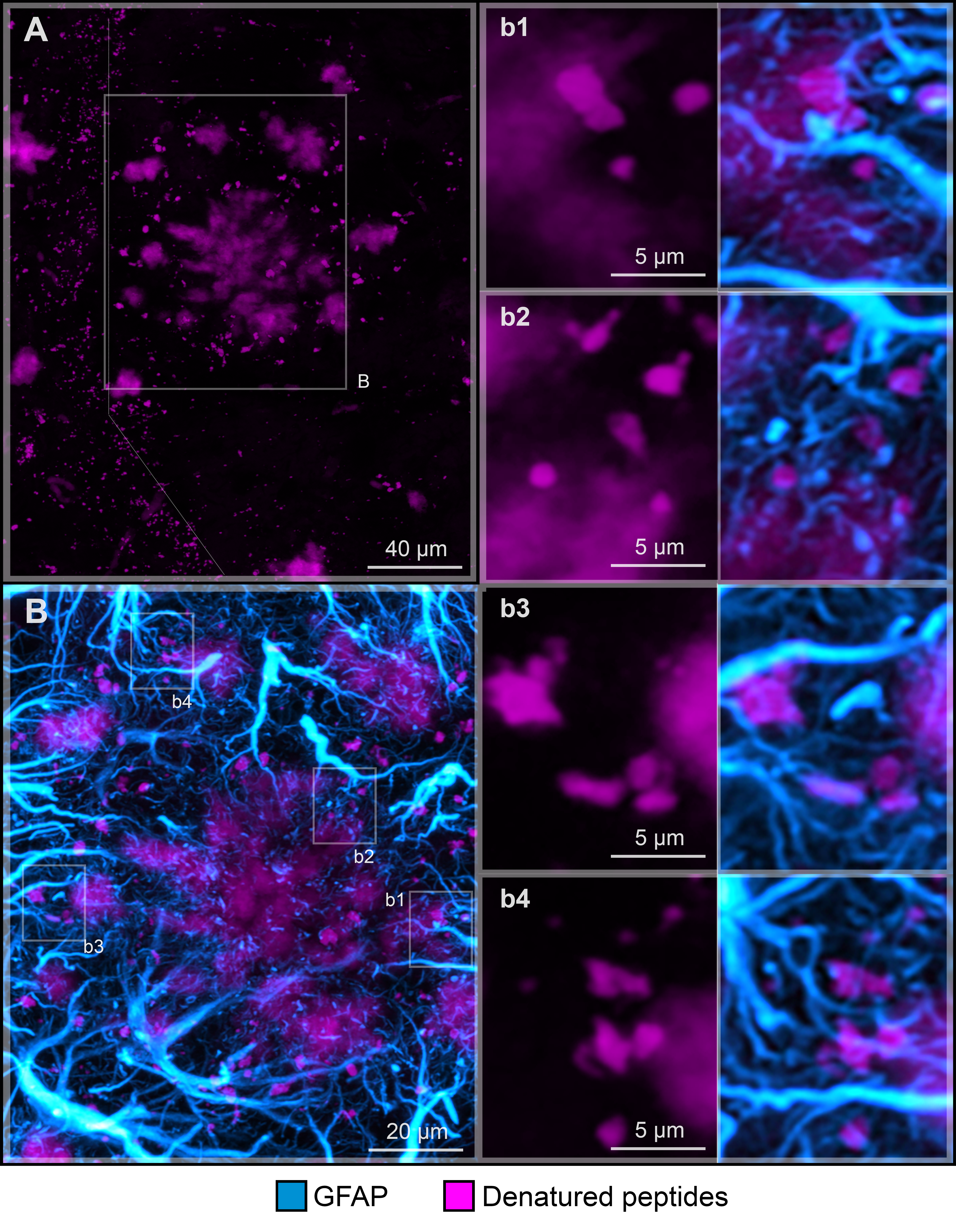

Supplement: Supplementary file 1 [file cells-12-02258-s001.zip › Supplementary figure S3.tif]

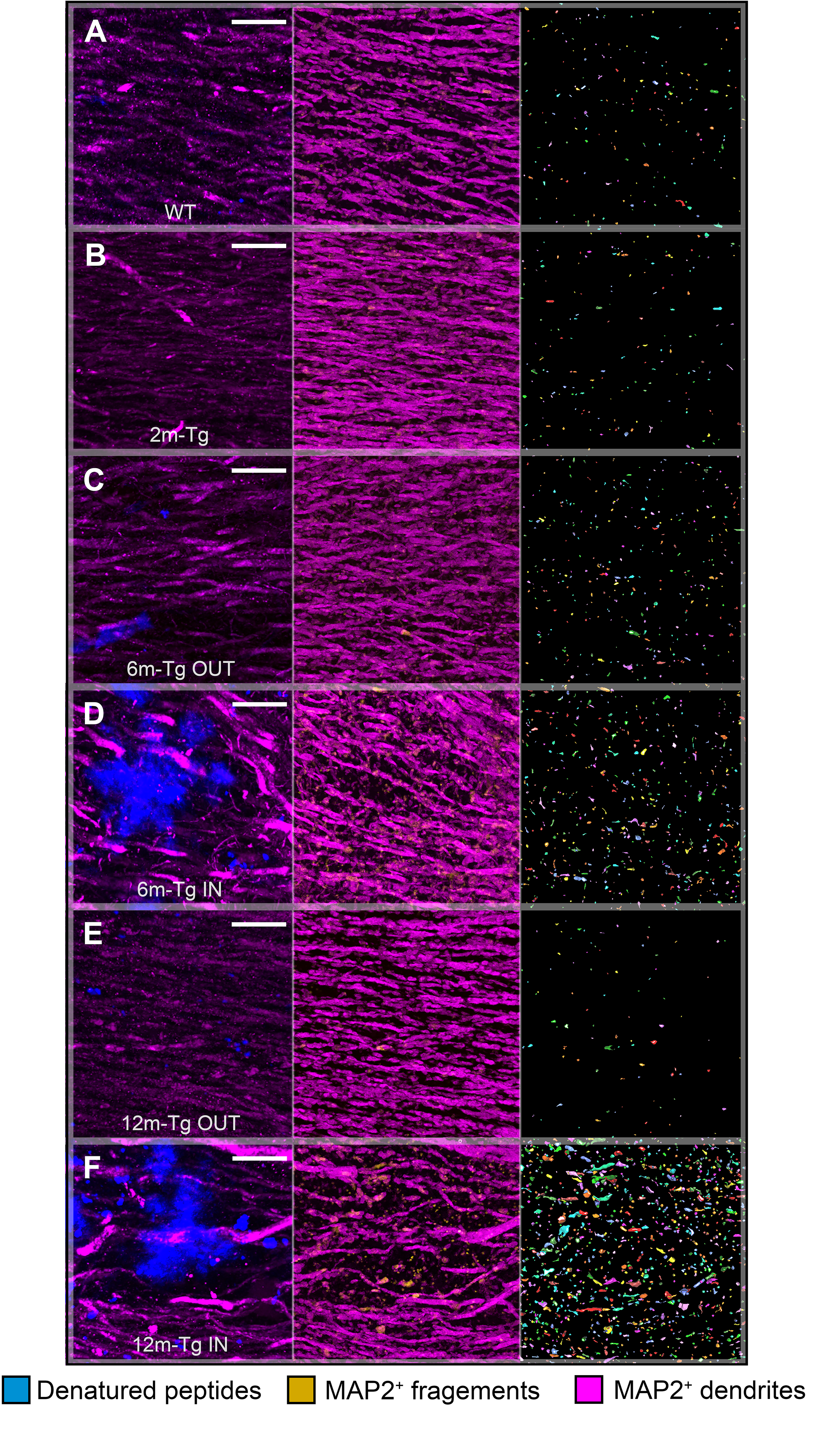

Supplement: Supplementary file 1 [file cells-12-02258-s001.zip › Supplementary figure S4.tif]
